# Beyond elimination: Assessing healthcare access, knowledge, attitudes, and practices regarding leprosy among communities in Lilongwe and Balaka districts of Malawi

**DOI:** 10.1371/journal.pntd.0014561

**Published:** 2026-07-17

**Authors:** Nathan Singano, Adriano Focus Lubanga, Joshua Kacheyo, Akim Bwanali, Leah Zuze, Steward Mudenda, Samuel Mpinganjira, Victor Mithi, Andrew M. Phiri, Chisoni Mumba

**Affiliations:** 1 Clinical Research Education and Management Services (CREAMS), Anderson House, Lilongwe, Malawi; 2 Department of Disease Control, School of Veterinary Medicine, University of Zambia, Lusaka, Zambia; 3 Malawi University of Science and Technology, Ndata School of Climate and Earth Sciences, Earth Sciences Department, Limbe, Malawi; 4 Zomba Central Hospital, Zomba, Malawi; 5 Department of Pharmacy, School of Health Sciences, University of Zambia, Lusaka, Zambia; 6 Kamuzu University of Health Sciences, Mahatma Ghandi Campus, Blantyre, Malawi; 7 University of Washington, Seattle, Washington, United States of America; 8 School of Global Health, University of Global Health Equity, Butaro, Rwanda; 9 Department of Clinical Studies, School of Veterinary Medicine, University of Zambia, Lusaka, Zambia; London School of Hygiene and Tropical Medicine, UNITED KINGDOM OF GREAT BRITAIN AND NORTHERN IRELAND

## Abstract

Although Malawi achieved national elimination of leprosy in 1994, new cases continue to be reported in several districts, including individuals presenting with Grade 2 disabilities, indicating delayed diagnosis and possible ongoing transmission. We assessed community knowledge, attitudes, and practices (KAP) regarding leprosy and examined geographic access to leprosy services in two endemic districts of Malawi. We conducted a community-based cross-sectional survey among 334 participants in Balaka and Lilongwe districts. KAP scores were calculated using composite indices, and multivariable regression models identified associated demographic factors. Spatial analysis was performed to estimate straight-line distance and travel time between residences of persons affected by leprosy and designated treatment facilities using proximity and road network analysis. Overall knowledge of leprosy causes and transmission was low, and negative attitudes were common. Higher educational attainment was independently associated with favourable knowledge and preventive practices. Spatial analysis showed that all affected individuals resided beyond a 5 km service catchment area. In Lilongwe, distances ranged from 12–15 km with estimated travel times of 10–40 minutes, while in Balaka distances ranged from 15–50 km with travel times of 45–80 minutes. These findings demonstrate that national elimination has not translated into equitable community awareness or physical access to care. Addressing post-elimination vulnerabilities through decentralised services, strengthened community education, and improved geographic accessibility will be essential to achieving zero leprosy.

## Author summary

Malawi achieved national elimination of leprosy over two decades ago; however, new cases and disability continue to be detected in several districts. We conducted a community survey to assess knowledge, attitudes, and practices related to leprosy and combined this with spatial analysis to evaluate access to treatment facilities. We found low community knowledge, persistent stigma, and substantial geographic barriers to care. Many individuals affected by leprosy lived far from specialised health services. These findings show that elimination status alone does not guarantee equitable access or sustained awareness. Continued community engagement and decentralised service delivery are essential to prevent delayed diagnosis and support global efforts toward zero leprosy.

## 1. Introduction

Leprosy is a neglected tropical disease caused primarily by *Mycobacterium leprae*, a bacterium that mainly affects the skin and peripheral nerves [1]. It is transmitted via aerosols during prolonged close contact with untreated individuals [2]. Without timely diagnosis and treatment, leprosy can lead to permanent nerve damage, visible deformities, long-term disability, and profound social consequences driven by stigma and exclusion [3]. According to the latest global data, 172,717 new leprosy cases were reported in 2024, with the majority occurring in the South-East Asia region, followed by America and Africa. Africa recorded one of the highest rates of severe disease, with a Grade 2 Disability (G2D) rate of 2.3 per million, and notably, almost half of new child G2D cases globally were reported from the African region [4].

The epidemiological profile of leprosy in Malawi reflects a complex post-elimination landscape. Although the country achieved national elimination status in 1994 [5], this does not mean eradication of the disease, as persistent transmission remains evident in several districts. Leprosy services in Malawi remain partially decentralised and are coordinated through designated referral facilities: Kamuzu Central Hospital and Bwaila District Hospital in Lilongwe, and Balaka District Hospital in Balaka. Smaller health centres generally refer suspected or complex cases to these main facilities for confirmation, treatment initiation, and follow-up.

As of 2024, Malawi reported a prevalence of 0.34 per 10,000 population, corresponding to 734 new cases among approximately 21 million people [4]. However, some districts continue to exceed the World Health Organisation elimination threshold of one case per 10,000 population. Longitudinal trends show fluctuating increases in case detection since 2018, driven largely by high-endemic districts. The continued occurrence of cases among children and individuals presenting with Grade 2 disability suggests delayed diagnosis and ongoing transmission. Treatment completion remains suboptimal at approximately 58%, despite the WHO recommendation of multidrug therapy (MDT) consisting of rifampicin, dapsone, and clofazimine to interrupt transmission and prevent complications [6]. These patterns highlight persistent gaps in community awareness, timely diagnosis, and access to care, particularly in geographically isolated settings where physical barriers limit healthcare access. Addressing such inequities aligns with Sustainable Development Goal 3, which emphasises universal health coverage and equitable access to essential health services [7].

Despite these challenges, post-elimination leprosy often receives less attention than competing public health priorities [8]. Reduced visibility can weaken surveillance, decrease community awareness, and delay case detection. Recognising these risks, the Global Leprosy Strategy 2021–2030 introduced a “Beyond Elimination” paradigm, shifting focus from prevalence-based targets toward the goal of zero leprosy [2]. This strategic shift emphasises the need for strong post-elimination surveillance and subnational, community-driven approaches to sustain progress. However, as noted by Muula, implementation of these principles remain incomplete in Malawi [9]. Limited disease knowledge, persistent stigma, and reduced access to skilled healthcare personnel continue to discourage early care-seeking and delay diagnosis.

Although national elimination indicators suggest success, they may conceal “post-elimination blind spots” where structural and social barriers continue to sustain disease risk. Understanding how knowledge, community perceptions, and access to healthcare interact in these settings is essential for preventing resurgence. Therefore, this study assessed community knowledge, attitudes, and practices (KAP) regarding leprosy in endemic districts of Malawi and examined geographic access to designated treatment centres using spatial analysis. This work aims to provide evidence to support more equitable post-elimination strategies aligned with the global goal of zero leprosy by integrating social and spatial dimensions.

## 2. Materials and methods

### 2.1 Ethical considerations

We obtained ethical approval from the National Health Sciences Research Committee (NHSRC) of Malawi (approval number: 24/03/4409) and administrative clearance from the Lilongwe and Balaka District Health Authorities. These approvals covered permission to access leprosy registers and to contact eligible persons affected by leprosy for recruitment, under strict confidentiality procedures. Access to identifiable records was restricted to authorised health staff, and the research team used the information only for initial contact and eligibility screening. We did not retain personal identifiers beyond participants’ leprosy status and district of residence, and all data were anonymised before analysis. Findings are reported in aggregate form to ensure that no individual participant can be identified.

We provided each participant with a one-to-one explanation of the study objectives, potential risks, and expected benefits in a private setting within their homes. We obtained verbal informed consent to accommodate varying literacy levels, and we required interviewers to sign a declaration on each questionnaire confirming that participants were adequately informed and voluntarily agreed to participate.

### 2.2 Study design, setting and health-service context

We conducted a community-based cross-sectional study in June 2024 in Balaka and Lilongwe districts of Malawi. The study comprised two linked components: a community knowledge, attitudes, and practices (KAP) survey and a spatial-access analysis among persons affected by leprosy who were actively enrolled in treatment.

According to the National Tuberculosis and Leprosy Elimination Programme, Balaka and Lilongwe were identified as leprosy-endemic districts because they have persistently reported case numbers above the WHO elimination threshold [1]. In the study districts, suspected leprosy cases may first present at lower-level health centres, but complex skin conditions and suspected leprosy are referred to district or referral facilities for clinical assessment, diagnosis, treatment initiation, and follow-up.

**Fig 1:**
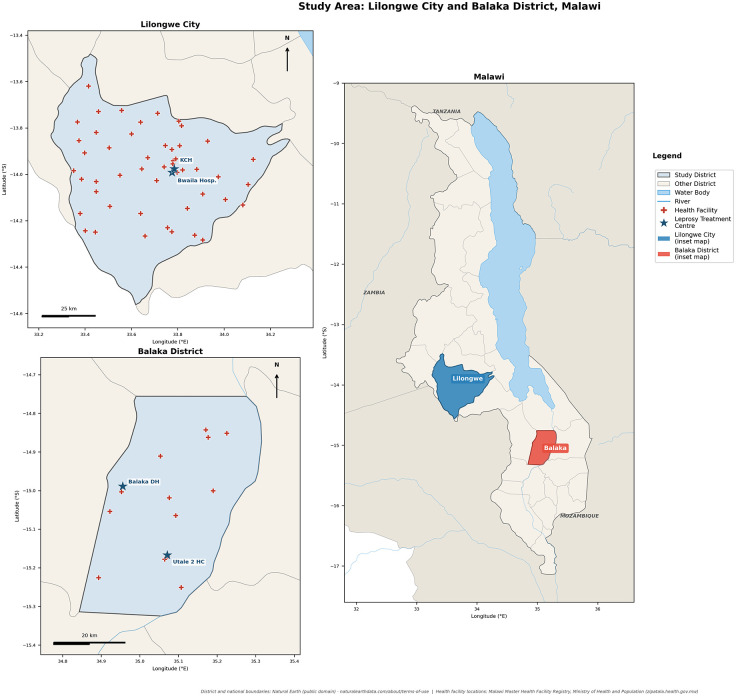
Map showing study sites. Stars indicate leprosy treatment centres. Plus symbols indicate other health facilities. The map was developed by the authors using ArcGIS version 10.3 (ESRI, Redlands, CA, USA), with basemap data from OpenStreetMap (OpenStreetMap contributors) and satellite imagery sourced from the USGS EarthExplorer platform (U.S. Geological Survey; https://earthexplorer.usgs.gov/.

Lilongwe City, the capital of Malawi, includes Kamuzu Central Hospital, a major referral facility, and Bwaila District Hospital, an important district-level facility (Fig 1). Both institutions play a role in leprosy diagnosis, treatment, and referral coordination. Balaka is a rural district in Malawi’s southern region. Balaka District Hospital serves as the main administrative and clinical coordination point for leprosy surveillance and management in the district (Fig 1). The Utale II Health Centre, also known as the Utale Leprosarium, was originally established as a leprosy clinic during the epidemic. It now functions as a regular health centre and depends on the District Health Office for technical support and coordination. The original settlement has since evolved into a permanent village inhabited by leprosy survivors and their descendants.

### 2.3 Sample size estimation and sampling procedure for the survey

We calculated a sample size of 384 community participants using the Cochran formula, assuming a 50% prevalence for the primary outcomes (knowledge and stigma) and a 5% margin of error. We split this target equally between the two districts to ensure balanced comparative power between the urbanised context of Lilongwe and the rural endemic setting of Balaka.

We defined the sampling frame as communities that were actively reporting leprosy cases to the Lilongwe and Balaka District Health Offices (DHOs). The primary sampling units (PSUs) were villages in Balaka and neighbourhoods in Lilongwe, selected based on their history of diagnosed leprosy cases. Within these PSUs, we used households as secondary sampling units. We enrolled participants using simple random sampling at the household level, and when multiple eligible adults were present, we randomly selected one participant. Eligibility criteria included being aged 18 years or older, residing in the selected village, and providing informed consent.

### 2.4 Recruitment of persons affected by leprosy

Because leprosy cases were geographically dispersed and contact tracing was challenging, we used purposive sampling to recruit individuals who were actively enrolled in treatment during the data collection period. We conducted systematic phone screening to assess participant availability, and among those reachable, we continued recruitment until all available and eligible individuals within treatment registers were exhausted, yielding a final sample size of 11 participants.

We specifically included this group to obtain residential coordinates required for measuring physical access to specialised leprosy care. Inclusion criteria were residence within the study districts and active enrolment in leprosy treatment at the time of data collection. Active enrolment was defined as being registered and receiving multidrug therapy at a designated leprosy treatment facility at the time of data collection without treatment interruption or default. Consequently, we excluded individuals who resided outside the study areas or those who had defaulted from or completed treatment.

### 2.5 Data collection tools and procedures

We used a structured questionnaire (S1) adapted from the Perception Study Toolkit (PST) developed by the International Federation of Anti-Leprosy Associations (ILEP) [10]. The PST contains modules tailored to different respondent groups (community members, index cases, and persons affected by leprosy). For this study, only the items specific to community members were selected, in line with the toolkit’s guidance, rather than applying the full instrument in its original form. The final questionnaire integrated modules on demographics, KAP, and the Explanatory Model Interview Catalogue Community Stigma Scale (EMIC – CSS).

We prepared the structured questionnaire in English and translated it into Chichewa, the local language. We trained data collectors before fieldwork and observed each interviewer conducting at least five interviews during the pretest phase to assess consistency and readiness for actual data collection. A spontaneous recall approach was used, whereby response options were not read aloud; instead, interviewers mapped participants’ responses to predefined categories. We piloted the tool among 38 participants from a setting comparable to the study sites to evaluate clarity and functionality. Following the pretest, we refined the questionnaire by revising ambiguous items and improving clarity. We excluded pilot participants from the final analysis. The full questionnaire is provided in the supplementary file.

#### 2.5.1 Spatial data collection.

We collected residential geographic coordinates using handheld GPS devices. Coordinates were recorded as latitude and longitude values and immediately anonymised by replacing identifiable location data with a unique participant code before storage and analysis. We identified designated leprosy treatment facilities within both study districts through the district health offices and recorded their coordinates for use as reference points in the proximity analysis.

### 2.6 Data management and analysis

We entered questionnaire data into Microsoft Excel and cleaned the dataset using the filter function before exporting it to R for analysis. We analysed the data using R software (version 4.3.3, R Foundation for Statistical Computing, Vienna, Austria). We generated descriptive statistics to summarise participant characteristics.

We calculated Knowledge, Attitudes, and Practices (KAP) scores using a composite scoring approach described by Urgesa et al. [11]. We assigned a score of 1 to correct responses and a score of 0 to incorrect or “don’t know” responses. Because KAP scores were not normally distributed, we categorised participants into binary outcomes using the median as the threshold. Participants scoring at or above the median were classified as having favourable knowledge and practices.

We conducted bivariate and multivariable analyses to examine factors associated with KAP outcomes. We initially used Pearson’s chi-square tests to screen for associations between demographic variables and KAP outcomes, considering p-values < 0.05 statistically significant. We included variables with p < 0.20 in multivariable models to identify independent predictors.

We analysed knowledge and practice outcomes using binary logistic regression. We categorised attitudes into three levels (negative, neutral, and positive) using tertile cut-offs (33rd and 67th percentiles) and analysed them using binary logistic regression. We selected final models using backward stepwise procedures based on likelihood ratio testing, with statistical significance set at p < 0.05.

#### 2.6.1 Spatial analysis.

We analysed spatial data using ArcGIS software (version 10.8, Esri, Redlands, CA). We conducted proximity analysis by calculating straight-line distances between participant residences and the nearest leprosy-designated health facilities. We further performed network analysis to estimate travel times along existing road networks We created 5-kilometre buffer zones around each designated facility to define approximate service catchment areas, in accordance with the WHO-recommended distance threshold for geographic accessibility to health facilities, which has been applied in previous spatial analyses conducted in Malawi and across sub-Saharan Africa [12–14]. We assessed whether participant residences fell within or outside these zones.

## 3. Results

### 3.1 Socio-demographic characteristics

A total of 334 participants were recruited, corresponding to an overall response rate of 87%. Non-participation was primarily attributed to absence at the time of household visits or time constraints rather than refusal to discuss leprosy.

Among participants, 61% were female and 49% were younger than 36 years. Educational attainment varied, with 52% having completed primary education and 34% having reached secondary education. Unemployment levels were high, with 63% of participants not engaged in formal employment. In addition, 74% reported earning less than USD 1.50 per day.

### 3.2 Knowledge regarding leprosy

Knowledge was assessed using 13 questions covering the cause, symptoms, and transmission of leprosy. Most participants (93%) reported having previously heard of leprosy; however, despite this familiarity, knowledge of the aetiology of leprosy was strikingly poor. Only 4% of participants correctly identified leprosy as a bacterial infection, while the vast majority (95%) reported not knowing the cause. Nearly three-quarters of participants (74%) did not know how leprosy is transmitted. Of those who responded, 17% correctly identified prolonged close contact with an untreated individual as the route of transmission.

Recognition of early signs and symptoms was also largely absent. More than half of participants (56%) did not know the early signs of leprosy. Among those who did respond, skin changes were the most recognised feature, with 23% selecting pale or reddish-brown patches on the skin. Neurological features were less frequently identified, with numbness in the hands and feet selected by 14% and loss of feeling in some areas of the skin by 10%. Muscle weakness was identified by only 3% of participants. Awareness of leprosy control initiatives was limited. Only 5% of participants reported awareness of government programmes aimed at raising leprosy awareness, and 8% were aware of Single-Dose Rifampicin (SDR) as a preventive intervention for contacts.

The mean knowledge score was 48.8 (SD ± 15.1), with a median of 50.0 (IQR: 42.9–57.1). Based on the median cut-off, 61% of participants were classified as having low knowledge.

Significant differences in knowledge levels were observed between districts and across educational levels (p < 0.05). Differences were also observed across occupational groups, with a higher proportion of unemployed participants classified as having low knowledge (Table 1).

**Table 1:** Knowledge distribution across demographics.

Knowledge					
Demographic Variables		Low	High	*χ* ^2^	*p* Value
		N (%)	N (%)		
Overall		205 (61)	131 (39)		
Gender	Female	130 (63)	75 (37)	1.276	0.25
	Male	75 (43)	56 (57)		
Age	18 – 24 years	52 (66)	27 (34)	4.436	0.49
	25 – 35 years	57 (65)	30 (35)		
	36 – 45 years	40 (61)	25 (39)		
	46 – 55 years	23 (52)	27 (48)		
	56 – 65 years	21 (57)	16 (43)		
	>65 years	12 (50)	12 (50)		
Educational level	<Secondary	131 (66)	69 (34)	4.184	**0.04**
	≥Secondary	74 (54)	62 (46)		
Marital status	Single	53 (59)	37 (41)	0.233	0.63
	Married	152 (62)	94 (38)		
District	Balaka	86 (55)	70 (45)	4.238	**0.04**
	Lilongwe	119 (66)	61 (34)		
Occupation	Unemployed	140 (65)	74 (35)	4.816	**0.03**
	Employed	65 (53)	57 (47)		
Daily income	<MWK 2500 (USD 1.50)	155 (65)	95 (32)	0.401	0.53
	≥MWK 2500 (USD 1.50)	50 (58)	36 (42)		

### 3.3 Attitudes toward leprosy

Attitudes were assessed using 16 questions examining perceptions and behaviours toward persons affected by leprosy. The median attitude score was 23.5 (IQR: 17.6–29.4). Based on tertile categorisation, 53% of participants were classified as having negative attitudes, 26% as neutral, and 21% as positive.

Indicators of stigma were common. Seventy-one percent of participants agreed that people with leprosy pose a threat to public health, while 35% agreed that leprosy causes shame or embarrassment within communities. Additionally, 56% reported that community members would avoid individuals affected by leprosy, including avoiding shared meals or purchasing goods from them.

Socioeconomic perceptions were also reported: 69% believed that a diagnosis of leprosy would make it difficult to find employment, and 48% perceived that it would create barriers to marriage. At the same time, 79% strongly agreed that open discussion about leprosy could reduce stigma, and 94% strongly agreed on the importance of showing compassion toward affected individuals.

Attitude levels varied significantly by gender, district, and daily income (p < 0.05). Female participants were more likely to report negative attitudes (58%) compared to males (46%). Participants from Lilongwe had a higher proportion of negative attitudes (60%) than those from Balaka (48%)(Table 2).

**Table 2:** Attitude distribution toward leprosy across demographic characteristics.

Attitude							
Demographic Variables			Negative	Neutral	Positive	*χ* ^2^	*p* Value
			N (%)	N (%)	N (%)		
Overall			179 (53)	87 (26)	70 (21)		
Gender		Female	119 (58)	41 (20)	45 (20)	9.617	**0.01**
		Male	60 (46)	46 (35)	25 (19)		
Age		<36 years	95 (57)	40 (24)	31 (19)	2.106	0.36
		≥36 years	84 (49)	47 (27)	39 (23)		
Educational level		<Secondary	117 (59)	47 (23)	36 (18)	5.530	0.06
		≥Secondary	62 (45)	40 (29)	34 (25)		
Marital status		Single	47 (52)	18 (20)	25 (28)	4.520	0.11
		Married	132 (54)	69 (28)	45 (18)		
District		Balaka	86 (48)	48 (27)	46 (25)	6.438	**0.04**
		Lilongwe	93 (60)	39 (25)	24 (15)		
Occupation		Unemployed	121 (57)	52 (24)	41 (19)	2.553	0.28
		Others	58 (47)	35 (29)	29 (24)		
Daily income		<MWK 2500 (USD 1.50)	144 (58)	58 (23)	95 (32)	7.418	**0.02**
		≥MWK 2500 (USD 1.50)	35 (41)	29 (34)	22 (25)		

### 3.4 Practices related to leprosy

Practice was assessed using 10 questions measuring health-seeking behaviours and preventive actions. The median practice score was 81.8 (IQR: 72.7–90.9), with 66% of participants classified as having good practice levels.

Most participants reported engaging in preventive and hygiene-related practices. Seventy-nine percent indicated that they frequently washed their hands after close contact with others, while 74% reported consistently covering their mouth and nose when coughing or sneezing. Additionally, 94% stated that they regularly encouraged good personal hygiene within their households.

Avoidance behaviours were also reported, with 72% indicating that they avoided close contact with individuals showing visible skin lesions or coughing symptoms when possible. Willingness to participate in health promotion activities was high, with 91% expressing readiness to participate in leprosy education programmes and 99% agreeing that schools should teach children about leprosy. No statistically significant differences in practice scores were observed across demographic categories.

### 3.5 Factors associated with Knowledge, Attitude, and Practice

A backwards stepwise binary logistic regression model was utilised to identify predictors of knowledge, attitudes, and practices (KAP) regarding leprosy. The final models retained education, gender and district as significant predictors of KAP outcomes (Table 3). Participants from Balaka district had 1.7 times the odds of having higher knowledge scores compared to participants from Lilongwe (OR = 1.7, 95% CI: 1.07–2.6, p = 0.024). Similarly, participants with secondary education or higher (OR = 1.8, 95% CI: 1.08–2.7, p = 0.025) were nearly twice as likely to demonstrate greater knowledge compared to those with only primary education or less. Females (OR = 2.1, 95% CI: 1.08–4.0, p = 0.028) were more likely to show positive attitudes towards leprosy compared to males. Education again played an important role in shaping practice; those with secondary education or higher were 1.6 times more likely to report better preventive practices compared to participants with only primary education (OR = 1.6, 95% CI: 1.1–2.7, p = 0.046).

**Table 3:** Factors associated with knowledge, attitudes, and practices regarding leprosy.

Variable	Odds ratio	95% CI	*p*-value	Outcome
**District**				**Knowledge**
Lilongwe	Ref			
Balaka	1.7	1.07 – 2.6	0.024*	
**Education**				
≤ primary level	Ref			
≥ secondary level	1.8	1.08 – 2.7	0.025*	
**Gender**				**Attitude**
Male	Ref			
Female	2.1	1.08 – 4.0	0.028*	
**Age**				**Practice**
< 36 years	Ref			
≥ 36 years	1.6	0.9 – 2.5	0.053	
**Education**				
≤ primary level	Ref			
≥ secondary level	1.6	1.1 – 2.7	0.046*	

* = statistical significance at p <0.05.

### 3.6 Access to leprosy healthcare facilities

Of the 11 participants included in the spatial analysis, 3 were from Lilongwe and 8 were from Balaka district. Five-kilometre buffer zones were created around designated health facilities to approximate reasonable access to leprosy services consistent with the WHO recommendation that populations should reside within 5 km of a health facility for adequate geographic access to care

**Fig 2:**
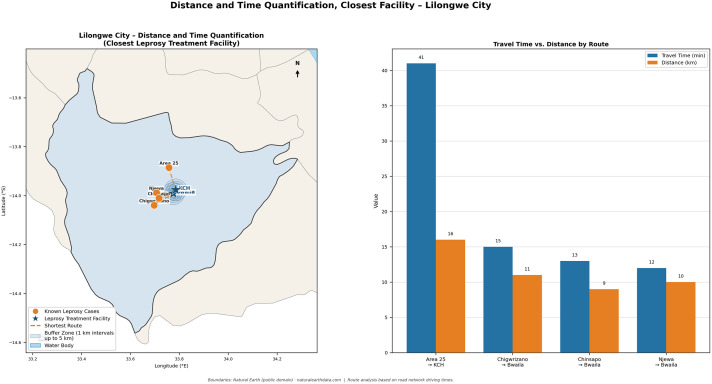
Distance and travel time from participant residences to leprosy treatment facilities in Lilongwe. The map was developed by the authors using ArcGIS version 10.3 (ESRI, Redlands, CA, USA), with basemap data from OpenStreetMap (OpenStreetMap contributors) and satellite imagery sourced from the USGS Earth Explorer platform (U.S. Geological Survey; https://earthexplorer.usgs.gov/.

All affected participants resided outside the 5-km buffer zones, indicating that they were located beyond the immediate catchment areas of designated leprosy services. In Lilongwe City, participants were located between 12 km and 15 km from the nearest treatment facility, with estimated driving times ranging from 10 to 40 minutes (Fig 2).

**Fig 3:**
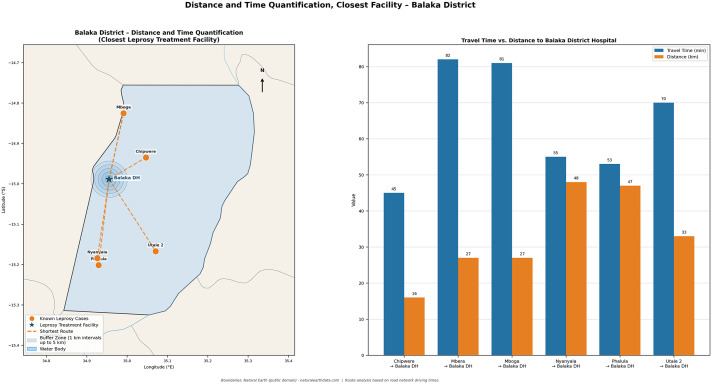
Distance and travel time from participant residences to leprosy treatment facilities in Balaka. The map was developed by the authors using ArcGIS version 10.3 (ESRI, Redlands, CA, USA), with basemap data from OpenStreetMap (OpenStreetMap contributors) and satellite imagery sourced from the USGS Earth Explorer platform (U.S. Geological Survey; https://earthexplorer.usgs.gov/.

In Balaka District, participants were located between 15 km and 50 km from the nearest treatment facility, with estimated driving times ranging from 45 to 80 minutes (Fig 3).

## 4. Discussion

This study identified low community knowledge of leprosy causes and transmission alongside persistent stigma and substantial barriers to healthcare access, demonstrating that elimination status at national level does not necessarily translate into adequate community-level preparedness or equitable service delivery. The coexistence of low knowledge, negative attitudes, and geographic inaccessibility suggests that post-elimination settings may remain vulnerable to delayed case detection, even when prevalence indicators appear favourable.

Knowledge levels differed significantly between districts, with participants in Balaka showing higher knowledge compared with those in Lilongwe. Several contextual factors may explain this pattern. Continued visibility of leprosy through the historical presence of the Utale Leprosarium Centre likely sustains community awareness and informal exposure to disease-related information. In contrast, urban and peri-urban settings such as Lilongwe may experience reduced disease visibility due to dispersed populations, competing health priorities, and weaker community-level health engagement. This finding highlights an important but often overlooked phenomenon in post-elimination contexts: declining visibility of a disease may unintentionally reduce public awareness, potentially weakening early recognition and care-seeking behaviours.

The low overall knowledge scores observed in this study align with previous findings from Malawi [5] and studies conducted in India [13,14] and Nigeria [15], where misconceptions about transmission, causes, and treatment remain common. Importantly, awareness of government leprosy initiatives and preventive interventions such as single-dose rifampicin, was extremely low. This may indicate a disconnect between programmatic activities and community outreach rather than an absence of interventions themselves. The high willingness among participants to engage in awareness programmes suggests that communities remain receptive to information when it is available, implying that current gaps are primarily related to communication reach and delivery mechanisms. This interpretation suggests that strengthening risk communication strategies may yield rapid improvements without requiring major structural changes.

Education emerged as a consistent predictor of both knowledge and preventive practices. Individuals with higher educational attainment were more likely to demonstrate favourable knowledge and behaviours, supporting evidence from Malawi and other endemic settings that education influences health literacy and reduces reliance on misconceptions [16–18]. Beyond individual understanding, education may also shape how communities interpret visible symptoms and respond to affected individuals, thereby influencing stigma dynamics and treatment-seeking pathways. This reinforces the importance of integrating leprosy education into broader health promotion and school-based programmes rather than relying solely on vertical disease campaigns.

The high practice scores observed reflect general hygiene behaviours that are incidentally protective against leprosy, rather than informed, disease-specific preventive actions. This distinction is important because good hygiene alone does not address the substantial knowledge gaps identified in this study; individuals who do not recognise the early signs of leprosy or understand its transmission are less likely to seek timely care or support affected persons appropriately. The elevated practice scores may also reflect the lasting influence of COVID-19 public health messaging, which substantially increased awareness of respiratory and contact hygiene in many communities.

Stigma remained a prominent feature of community perceptions. A substantial proportion of participants associated leprosy with shame, social exclusion, and reduced economic or marital opportunities. Such beliefs can operate through multiple pathways to delay diagnosis: individuals may conceal early symptoms, families may discourage disclosure, and community members may avoid affected persons, all of which reduce opportunities for timely referral. Similar patterns have been described in Ethiopia and other endemic settings [11]. Importantly, the coexistence of stigma and high compassion-related responses suggests that attitudes may be complex rather than uniformly negative. While participants expressed empathy toward affected individuals, underlying fears of contagion and social consequences remained strong. This indicates that stigma reduction strategies may need to move beyond awareness campaigns alone and incorporate community dialogue and social inclusion approaches, as highlighted in previous research [19].

The geospatial analysis provides descriptive insight into the travel burden faced by individuals accessing leprosy treatment. All affected participants lived outside the defined service catchment areas, and in some cases travelled considerable distances to access treatment. While these findings do not permit conclusions about whether distance prevents individuals from initially seeking care, given that the sample comprises only those already attending services, they do raise important questions about barriers to treatment retention and completion. Particularly, in populations where most households survive on less than USD 1.50 per day. Sustained attendance over a multi-year treatment course with together with such distances may represent significant financial and logistical burden. Travel-related barriers can discourage early presentation, particularly for conditions such as leprosy with slow onset and limited early symptoms. Similar transportation and access challenges have been reported in Malawi [20,21], reinforcing the importance of strengthening the capacity of frontline health workers to recognise and refer suspected leprosy cases may therefore be more critical than expanding specialist facility catchment areas, and would align with how care is realistically accessed in this setting.

An important interpretation emerging from these findings is the interaction between social and spatial barriers. Low knowledge may delay recognition of symptoms, stigma may discourage disclosure, and long travel distances may further postpone healthcare utilisation. Together, these factors can create a reinforcing cycle that results in late diagnosis and increased disability. This combined effect highlights the need to view leprosy not only as a biomedical condition but also as a socially and structurally mediated health issue.

These findings also raise broader questions regarding how elimination is measured. National elimination indicators primarily reflect prevalence thresholds and may not capture inequities in access or community awareness. As highlighted by the Sustainable Development Goal framework and universal health coverage targets [7], equitable access remains central to effective health systems. The continued existence of access barriers despite elimination status suggests that surveillance systems may benefit from incorporating social and spatial indicators in addition to epidemiological measures. Consistent with the observations of Peters et al. [22], geographic accessibility continues to play a critical role in healthcare utilisation in low-resource settings.

The study additionally suggests that post-elimination periods may represent a phase of programmatic vulnerability. Reduced disease visibility and shifting public health priorities may unintentionally weaken community engagement and early detection systems. Sustained investment in education, community outreach, and frontline healthcare worker capacity may therefore be essential not only for maintaining low prevalence but also for preventing resurgence.

The findings demonstrate that achieving zero leprosy requires a transition from elimination-focused metrics toward approaches that address social perceptions, healthcare accessibility, and community empowerment. Integrating decentralised services, strengthening community-based awareness programmes, and embedding leprosy within broader primary healthcare systems may provide more sustainable long-term control in post-elimination settings.

### 4.1 Limitations of the study

This study has several limitations that should be considered when interpreting the findings. First, the cross-sectional design limits the ability to establish causal relationships between demographic factors and KAP outcomes. Associations identified in the analysis should therefore be interpreted as correlational rather than causal.

Second, although the sample size was sufficient for the planned analyses, the final number of participants was slightly lower than the calculated target. This may have reduced statistical power for detecting smaller differences across some subgroups.

Third, the subgroup of participants affected by leprosy included in the spatial analysis was relatively small and exclusively from individuals already accessing leprosy services. As such, the findings reflect travel burden among treatment attendees rather than access barriers among the broader affected population. While these data provided useful insights into geographic access barriers, the findings may not represent all individuals affected by leprosy within the study districts. Future studies with larger geographic samples could provide more comprehensive spatial analysis by incorporating spatial data from non-attending individuals and general health facility utilisation patterns would allow more robust conclusions regarding access as a barrier.

Fourth, geographic accessibility was assessed using distance and estimated travel times based on road networks. These measures may not fully capture other important barriers such as seasonal road conditions, transportation costs, or household-level financial constraints, which may further influence healthcare utilisation.

Finally, KAP data were based on self-reported responses and may be subject to social desirability bias. Participants may have reported more favourable attitudes or practices than those enacted in everyday life.

Despite these limitations, the study provides valuable evidence on the interaction between knowledge, stigma, and access to care in a post-elimination context and contributes important insights to ongoing efforts to achieve zero leprosy.

## 5. Conclusion

Although Malawi achieved national elimination of leprosy decades ago, this study demonstrates that important social and structural barriers persist at the community level. Low knowledge of leprosy causes and transmission, continued stigma, and limited physical access to specialised services indicate that elimination status has not fully translated into adequate community-level knowledge and equitable access to diagnosis and treatment. These gaps may contribute to delayed diagnosis and continued risk of transmission in endemic districts.

The findings highlight the need to move beyond prevalence-based elimination indicators toward sustained post-elimination strategies that prioritise community engagement, health education, and decentralised service delivery. Strengthening awareness programmes and addressing stigma at community level, alongside improving access to diagnosis and treatment through frontline health systems, will be essential for promoting early case detection.

Achieving the goal of zero leprosy will require sustained investment in surveillance, health system strengthening, and socially responsive interventions that address the structural and behavioural factors shaping care-seeking. Integrating social and spatial considerations into post-elimination policies may help ensure that progress toward elimination translates into lasting and equitable disease control.
